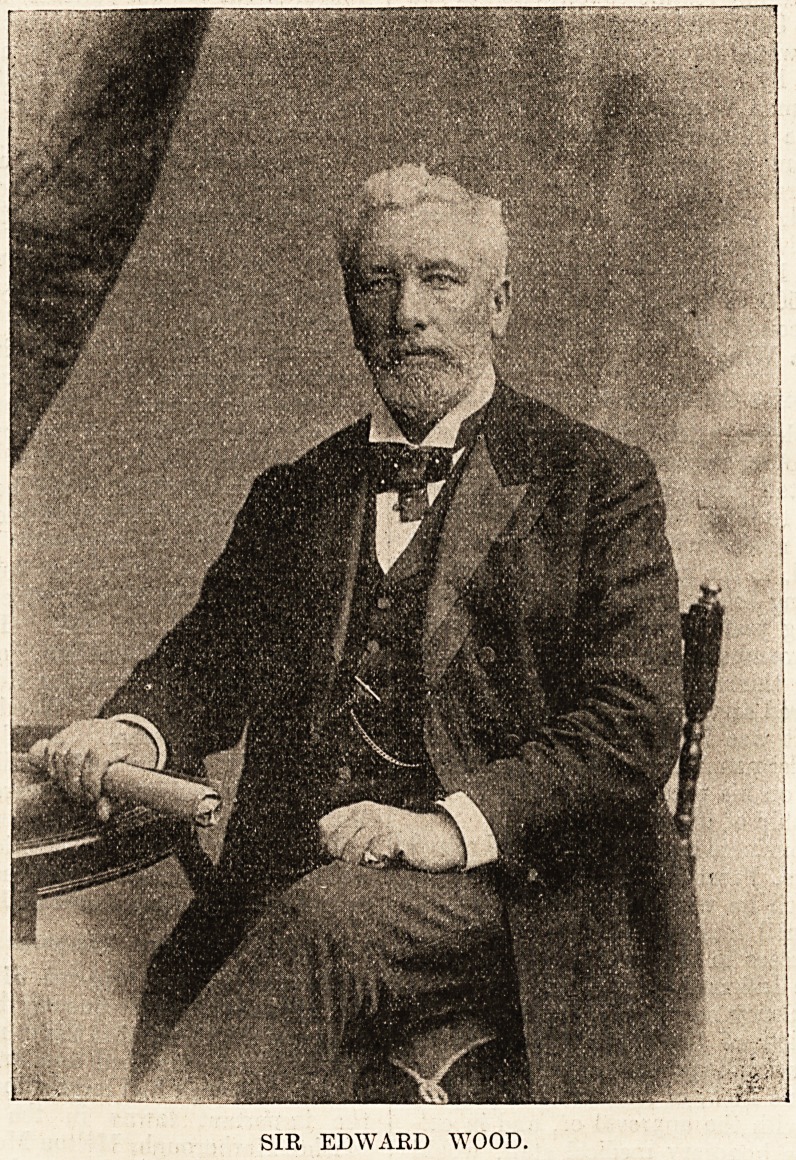# Eminent Chairman Series

**Published:** 1910-11-05

**Authors:** 


### SPECIAL INSTITUTIONAL ARTICLES.

### EMINENT CHAIRMAN SERIES.

### II.—SIR EDWARD WOOD, CHAIRMAN OF THE BOARD OF GOVERNORS OF THE LEICESTER INFIRMARY.

The great improvement and reconstruction scheme which has been carried out in connection with the Leicester Infirmary has brought into public prominence the personality of the Chairman of the Board, Sir Edward Wood.

First opened on September 11, 1771, the Infirmary has uninterruptedly continued its beneficent work—alleviative, remedial, curative. From time to time as the population increased (it was estimated to be 12,000 at the time of the foundation of the Infirmary, and is now 240,000) the institution has been enlarged and added to, but its original buildings remained practically unmodernised. Thus, until quite recent years, the Leicester Infirmary was an example of a type of hospital endeavouring to carry on modern warfare against sickness and disease with implements, some partially up to date, others obsolete.

SIR EDWARD WOOD.

It was not, therefore, surprising that the honorary medical and surgical staff in the year 1900 called attention to serious defects which existed, and urged the Board to remodel the whole drainage system, as well as some of the structure, and provide extensive additions and improvements necessitated by the largely increased population.

The first step taken to give effect to these recommendations was the building of the Victoria Wing (opened in 1902), containing modern operating theatres, a new accident department, resident doctors' sitting and bed rooms, etc. It was during the construction of this building that Sir Edward Wood, or, as he then was, Alderman Wood, became closely associated with the Infirmary, with the result that a depressing outlook which at that time faced the Governors has given place to one of hopefulness and brightness.

The Mayor of Leicester (Alderman Lennard), son-in-law of Alderman Wood, had in the year 1902 inaugurated a Queen Victoria Commemoration Fund, by which a sum of £25,000 was raised as a Memorial to Queen Victoria. Just before the closing of the Fund Alderman Lennard died, and Alderman Wood undertook his son-in-law's Mayoral duties and brought the Commemoration Fund to completion.

The Governors of the Infirmary, being hard pressed for funds to pay for the construction of the wing they had just built, and being face to face with the fact that they would have to deplete the already insuffîcent income by a sale of investments, made an appeal to Alderman Wood and the Committee of the Queen Victoria Commemoration Fund for the allocation of a sum of money from the Mayor's Fund. The Governors' appeal was most sympathetically received, and Alderman Wood subsequently had the privilege of reporting that the Committee of the Fund had approved of the contribution of a sum of £12,500 to the Infirmary mainly for the new wing, on condition that it should be named the Queen Victoria Wing.

### Mr. Wood becomes Chairman of the Board.

This wing completed, attention was prominently attracted to many other serious defects of structure, and the medical staff pressed for further additions and improvements to bring the institution to a state of efficiency and modernity. The way was not however clear, and, to the great loss of the institution, Mr. T. Fielding Johnson, J.P., felt compelled, owing to advancing years, to resign his chairmanship and also his membership of the Board, which had continued for thirty-one years, during which time he had rendered services of great advantage to the institution. It was in these somewhat acute difficulties that Alderman Wood was, not altogether without misgiving on his part, persuaded to undertake the chairmanship of the Board.

With that keen business acumen which had raised the firm of Messrs. Freeman, Hardy and Willis, Limited, over which he was the presiding genius, from that of a small local concern to one of the best known businesses in the country, he at once realised that if the Infirmary was to be brought to a state of real efficiency no less a scheme than the entire reconstruction of the institution must be undertaken. He accordingly instructed the architect (Mr. S. Perkins Pick, of Leicester) to prepare a scheme which, with the approval of, and in collaboration with the honorary medical staff, would bring the institution up to date.

### The Reconstruction Scheme.

The following recommendations were submitted to the Board: (1) The entire reconstruction of the drainage system; (2) the enlargement of the ground area of the institution by the purchase of the site and cottages forming practically one side of Knighton Street adjoining the institution; (3) the removal of the old boiler-house from its site near the hospital, and the reconstruction of a new boiler-house, as well as steam disinfector and refuse destructor, on a site at the south-western extremity of the grounds; (4) the entire reconstruction of the heating arrangements by replacing the old calorifiers by the installation of modern machinery and equipment worked from a pump-room fitted with duplicate machinery forming part of the new boiler-house block; (5) the entire reconstruction of six very old wards in the N.E. and N.W. portions of the building and the erection of separate sanitary annexes detached from the wards; (6) the reconstruction of the kitchen and culinary department; (7) the removal of the old out-patients' department from the then position under the principal female medical ward to an entirely new site, and the erection of a larger and complete department, self-contained and separate from the Infirmary, at the S.E. side of the grounds.

This scheme was submitted as giving an absolute minimum of the necessary requirements, and the architect estimated the cost at £50,000.

### Raising the Funds.

The position was certainly not an enviable one for a new Chairman, but Alderman Wood threw himself with vigour and hopefulness into his task, and a meeting representative of all classes was called to consider the position. The Lord Lieutenant of the County (the Marquis of Granby, now the Duke of Rutland, and the President of the Infirmary) presided, and Alderman Wood having submitted the scheme, appealed for £50,000 to accomplish it. The amount seemed an impossible one; but Alderman Wood handed a list of contributions from his firm and from personal friends, which, with £5,000 from himself, totalled £12,500. In a moment doubt gave way to enthusiasm, and enthusiasm to generosity, and the meeting closed with the knowledge that Alderman Wood had successfully launched his scheme. Building operations were immediately commenced, until, one by one, the whole of the improvements were taken in hand, and the £50,000 raised and expended.

In 1906, to the keen satisfaction of the whole town and county, Alderman Wood received the honour of knighthood from the hands of King Edward.

### The Need for Further Enlargement.

During the carrying-out of the extensive improvements it had frequently been brought to the notice of the Board that the number of beds had for twenty years remained unaltered, notwithstanding that the borough had largely increased in size, that the important towns in the county—Hinckley, Market Harborough, Melton Mowbray—had largely increased in population, and consequently their demands on the County Hospital were greater, that increasing applications for admission came from the County of Rutland, that the conversion of some of the smaller county parishes into populous centres caused by the upspringing of numerous boot, hosiery, elastic web, and other factories, had increased the work of the institution, the pressure on the accommodation of which was of such a nature as to call for immediate remedy.

The winter of 1905 served to accentuate the great need for more beds, for in addition to some two hundred cases waiting admission, it had frequently to be reported that the average number of in-patients for many weeks together exceeded the number of beds, that even surgical cases had to be accommodated on couches in the wards, and that sometimes serious cases—cases of cancer, the only chance of relief for which lay in immediate operative treatment, had at times to await vacant beds. But in addition to the inadequacy of the bed accommodation for patients, another need had prominently presented itself, the provision of adequate accommodation for the nursing staff. The largely increasing work of the institution especially on the surgical side, coupled with the large reconstruction scheme which had been carried out, had necessitated an increase in the nursing staff. The accommodation for the former staff in the hospital buildings had been quite inadequate for some years, and the leasing of a complementary home at Aylestone on the outskirts of the borough had increased rather than diminished the anxieties of the Board and Miss Rogers, the lady superintendent. With the prospect of a large addition to the number of beds, the Board were face to face with two alternatives—either another large establishment must be created for a Nurses' Home by taking another house apart from the institution, or a new Nurses' Home sufficient for all purposes must be provided. The position was a serious one, large additional accommodation for patients was inevitable, and when provided could not be administered without an adequate Nurses' Home. The question arose as to which was the more vital and immediate need, and it was not without serious consideration that Sir Edward Wood and the Board decided in favour of the additional beds. Accordingly at the 134th anniversary meeting held on March 26th, 1906, which was largely attended by the Governors and subscribers, and presided over by the Viscount Churchill, G.C.V.O., a resolution was, after a statement by Sir Edward Wood as to the pressing need of additional beds, unanimously passed giving approval to the decision of the Board of Governors to provide accommodation for a hundred additional beds.

### Facing the Problem.

Sir Edward Wood lost no time in facing the great responsibilities—already £50,000 had been raised, and spent on the reconstruction scheme, and a further appeal must be made for a large sum.

Plans were submitted by the architect under the guidance of the honorary medical and surgical staff for a new wing capable of accommodating a hundred patients—ample provision being made for every modern feature of medical and surgical ward work.

The site chosen was that occupied by the disused fever house and the old out-patients' department, above which was the principal female medical ward, while the top story was occupied as bedrooms for the female domestic staff. The question then arose as to whether the conversion of the building into modern wards was from a constructive point of view expedient, and if expedient whether the cost would be considerably less than the entire demolition of the old building and the erection of a modern and efficient building on the site.

At the request of the Board the architect submitted an elaborate and well-considered report in which he pointed out that from a constructive point of view the conversion was possible, although he was of opinion that an entirely new ward would provide features of importance, which the conversion would not permit of. The cost of the former scheme he estimated at £15,400 and the latter £18,000. During the consideration of this report Sir Edward Wood had, however, been rendering yeoman and effective service, and when the Board met to consider the report, he was able to impart an announcement which was received with the utmost enthusiasm. Messrs. G. C. Oliver, J.P., C. F. Oliver, and H. B. Oliver, sons of the late Mr. George Oliver, J.P., a gentleman who had been closely associated with the business life of the town, had generously consented to provide a sum of £6,000 to build and equip a ward as a mark of affection and esteem for their father. This munificent sum, he reported would form a substantial nucleus for the provision of the additional accommodation, and if the Board were of opinion that it was in the interests of the Infirmary to build a new wing rather than reconstruct the old, he thought they might embark on the larger outlay. Guided by the success and well-founded optimism of the Chairman, the Board declared for the larger scheme, and Sir Edward Wood then announced that he was prepared to repeat his donation to the original reconstruction scheme by giving a second sum of £5,000. The Board rose after a long and enthusiastic sitting, the Chairman's munificence, enterprise, and success being the theme for the heartiest congratulation and keenest satisfaction.

### The Nurses' Home.

The new wards building was thus commenced under favourable auspices, but still further generous help was in store. One morning Mr. Samuel Odames—a gentleman closely associated with the business life of the town—presented himself at the institution before the meeting of the Weekly Committee, and after inspecting the wards of the Infirmary and of the Children's Hospital announced his intention to endow by testamentary gifts a bed in the Infirmary and cots in the Children's Hospital. This welcome announcement was intimated to Sir Edward Wood, who, with that keen business acumen which has shown itself in all his undertakings, foresaw a generous and unexpected benefactor, and at once threw out a challenge that, if a second ward were forthcoming on similar terms to that provided by the Messrs. Oliver in memory of their father, he would at once proceed with the scheme for the erection of a new Nurses' Home. Negotiations were entered into, many interviews held, and after some weeks Sir Edward Wood had the great pleasure of announcing an immediate offer by Mr. Odames to commemorate his eighty-fourth birthday by building and equipping a second ward, to be called after himself. This success was not in itself single, for the Chairman was also able to report other munificent promises of help, and by the time the new wing was completed he was able to say that not only had the whole cost been provided, but he estimated £6,000 would be in hand towards the Nurses' Home if the Board decided on its erection.

### THE NEW WING.

The new wards' wing—the foundation-stone of which was laid on February 27, 1907, by the Duke of Rutland—was opened on November 5, 1907, by H.R.H. Princess Louise, Duchess of Argyll, who was accompanied by the Duke of Argyll. A luncheon was given by the Chairman, with the gracious permission of Her Royal Highness, in the Mayor's rooms, among the guests being the Duchess of Rutland, the Viscount Churchill, and the contributors to the extension schemes. Subsequently Her Royal Highness declared the new wing open, and opened a bazaar in one of the wards. A general holiday was observed in the Borough, and so great was the demand for admission to the opening ceremony that hundreds who had purchased tickets got but a faint glance of Her Royal Highness, and still less of the formal opening. The bazaar, which was organised by the nursing staff, and by a Committee representing the industries of the town, led by Mr. J. A. Corah, J.P., proved the most successful ever organised in the town, and the total receipts amounted to £4,510. During the construction of the new wing, however, the necessity for yet another enlargement and improvement was brought into prominence.

The large scheme which had been carried out had made increased demands on the laundry, which it had proved impossible to meet, and it was readily perceived that, equipped as it was by machinery not of a modern type, the laundry would be quite incapable of successfully meeting the additional work which the new wing would entail. Reconstruction was therefore decided on, and plans were submitted not only for extensive additions, but for a modern and efficient plant, new drying rooms, etc. The total cost of these improvements was £2,700. The Board, by a unanimous decision, also decided to proceed with the erection of a modern and complete Nurses' Home, capable of accommodating 100 nurses, with adequate sitting rooms, reading and writing rooms, recreation room, and a separate bedroom for each nurse. The cost of building and furnishing was £24,000. This building was fully described in THE HOSPITAL of February 5, 1910, and the foundation-stone, which was laid on November 4, 1908, bears the following inscription:—

THIS STONE WAS LAID BY
MISS GERTRUDE A. ROGERS,
LADY SUPERINTENDENT OF THE INFIRMARY,
NOVEMBER 4, 1908.

The building was finally completed and opened on February 8, 1910, by Mrs. Fielding Johnson, the wife of the former Chairman, who himself gave £500 to the fund for its erection, the Chairman likewise giving an additional £500, while the family of the Chairman gave a portrait in oils of Sir Edward Wood, which is hung in the principal entrance, and will remain one of the institution's most treasured possessions. The Nurses' Home has been designated the "Edward Wood Home," as the Board were unwilling that the name of the Institution's greatest benefactor should be omitted from the handsome buildings, which by his enterprise make the Leicester Infirmary one of the best equipped of the provincial hospitals.

The entire cost of all these improvements and additional totals, in round figures, was £100,000, and it would be an obvious omission not to chronicle the fact that Sir Edward Wood's personal contributions have amounted to £12,500—one sovereign for every seven which have been raised and expended.

### THE WORK ACCOMPLISHED.

Great pride may not unnaturally be taken by the residents of the Borough and county in the County Hospital, which, by the magnificent help they have rendered, has been brought up to a modern standard of high efficiency, and will remain a lasting tribute to their philanthropy. Even now, however, a moderate judicious expenditure may yet accomplish improvements, as was recently pointed out by Sir Edward Wood at the last quarterly meeting of the governors and subscribers. He was, he said, sometimes asked whether they had done everything they intended to do. It was quite true that there was other work which might be done, but he thought it would be inadvisable at present to make an appeal for further money. He would, however, tell them quite frankly that several improvements were desirable, although not of absolute urgency.

The Accident Ward, built in 1862, was lacking in the best features of the newer wards. The floors were of white wood, the heating by open fires and chimneys, instead of by central stoves with underground flues, electric light might be introduced, and there were no balconies, which were now so desirable in a surgical ward, and it was doubtful if they could be provided as additions. The former Nurses' Home was now in disuse—its structure made remodelling difficult. The Children's Hospital needed enlargement, and could certainly be improved at a comparatively small outlay.

The Board could very advantageously spend another £30,000, although they were going to take the advice of a great statesman and "Rest and be thankful." He, however, outlined the scheme because he felt the Infirmary must look ahead in its administration, and he hoped some benefactor might be constrained to come forward in the service of the community with a noble offer to undertake these improvements, and so complete in its entirety a great scheme of reconstruction.

Side by side, however, with the raising of £100,000 for building purposes, the annual income of the Infirmary has been built up under the auspices of Sir Edward Wood, with good results. These mark an additional success as remarkable as it is gratifying to the community for which Sir Edward Wood has laboured with such self-denying and successful personal service.

## Figures and Tables

**Figure f1:**